# Review of Thermal and Physiological Properties of Human Breast Tissue

**DOI:** 10.3390/s22103894

**Published:** 2022-05-20

**Authors:** Jeantide Said Camilleri, Lourdes Farrugia, Sergio Curto, Dario B. Rodrigues, Laura Farina, Gordon Caruana Dingli, Julian Bonello, Iman Farhat, Charles V. Sammut

**Affiliations:** 1 Department of Physics, Faculty of Science, University of Malta, MSD 2080 Msida, Malta; lourdes.farrugia@um.edu.mt (L.F.); julian.bonello@um.edu.mt (J.B.); iman.farhat@um.edu.mt (I.F.); charles.v.sammut@um.edu.mt (C.V.S.); 2 Department of Radiotherapy, Erasmus MC Cancer Institute, University Medical Center Rotterdam, 3015 GD Rotterdam, The Netherlands; s.curto@erasmusmc.nl; 3 Department of Radiation Oncology, University of Maryland School of Medicine, Baltimore, MD 21201, USA; drodrigues@som.umaryland.edu; 4 Translational Medical Device Lab, National University of Ireland Galway, H91 TK33 Galway, Ireland; laura.frna@gmail.com; 5 Department of Surgery, Mater Dei Hospital, MSD 2090 Msida, Malta; gordon.caruana-dingli@gov.mt

**Keywords:** thermal properties, physiological properties, breast tissue, breast cancer, Pennes bioheat equation

## Abstract

Electromagnetic thermal therapies for cancer treatment, such as microwave hyperthermia, aim to heat up a targeted tumour site to temperatures within 40 and 44 °C. Computational simulations used to investigate such heating systems employ the Pennes’ bioheat equation to model the heat exchange within the tissue, which accounts for several tissue properties: density, specific heat capacity, thermal conductivity, metabolic heat generation rate, and blood perfusion rate. We present a review of these thermal and physiological properties relevant for hyperthermia treatments of breast including fibroglandular breast, fatty breast, and breast tumours. The data included in this review were obtained from both experimental measurement studies and estimated properties of human breast tissues. The latter were used in computational studies of breast thermal treatments. The measurement methods, where available, are discussed together with the estimations and approximations considered for values where measurements were unavailable. The review concludes that measurement data for the thermal and physiological properties of breast and tumour tissue are limited. Fibroglandular and fatty breast tissue properties are often approximated from those of generic muscle or fat tissue. Tumour tissue properties are mostly obtained from approximating equations or assumed to be the same as those of glandular tissue. We also present a set of reliable data, which can be used for more accurate modelling and simulation studies to better treat breast cancer using thermal therapies.

## 1. Introduction

Breast cancer is the most common type of cancer in the world, with an estimated incidence rate of 12.5%, which amounts to around 25% of all the cancer diagnoses in women [1]. Various modalities are utilised to treat breast cancer such as radiotherapy, chemotherapy, and hormone therapy. However, these treatments are not without their limitations, with distressing physical and psychological impacts on the patient. One emerging treatment for breast cancer is hyperthermia therapy (HT), where the tumour site is heated to temperatures between 40 and 44 ${}^{\circ }C$ for approximately 1 h [2]. The elevated temperature increases the tumour sensitivity to other cancer treatments such as radiotherapy and chemotherapy, enhancing their effectiveness. Such heating can be obtained using non-ionising electromagnetic energy such as radiofrequency (RF), microwaves (MWs), or ultrasound [3,4,5,6].

On a macroscopic scale, the increase in temperature within tissue causes blood perfusion to increase within the heated tissue, an effect that is temperature dependent [7]. This is a thermoregulation mechanism since the body tries to counteract the temperature increase by perfusing the heated region with blood at core temperature (around 37 ${}^{\circ }C$), making blood perfusion an effective heat sink that removes heat from the tumour and prevents surrounding healthy tissue being heated. Furthermore, hyperthermia also activates the immune system, in a process very similar to naturally induced fevers [8]. This blood perfusion increase from hyperthermia can improve the pathway between the tumour and the draining lymph nodes, thus attracting immune cells. The effect on the immune system varies depending on the duration of heating and the temperature reached [9].

In most studies involving thermal therapies, computational simulations of a numerical model are often used to investigate the heating generated within the tumour and surrounding tissue. The aim is to evaluate the performance of the heating device and assess the heating zone created to maximise energy deposition in the tumour site, while minimising the energy delivered to the surrounding healthy tissue.

These computational models are a critical tool in the designing stage of HT applicators, where optimisation adjustments and fine-tuning the design to a particular cancer site are implemented before building the first prototype. In the case of electromagnetic hyperthermia, computational simulations are performed to obtain the electromagnetic fields from the HT device. These results are then used to generate a temperature map within the tissue of interest, which is calculated through a heat transfer equation, where Pennes’ bioheat transfer equation is the most widely used [10].

After the design is optimised, the following step is to test the device on a physical phantom. In order to obtain and measure relevant temperature maps in phantoms, these physical models must mimic the human anatomy and include mixed materials that mimic tissue properties. While the dielectric properties of breast tissue have been extensively studied [11] and modelled [12] to be applied to computational and physical phantoms, the thermal properties of breast tissue have not been thoroughly investigated and compiled. Both testing scenarios (computational and physical phantoms) require accurate knowledge of the thermal properties of breast tissues to obtain clinically relevant thermal distributions.

Oftentimes, the thermal properties of breast tissue are approximated to those of other well-measured tissues such as muscle or fat. In some cases, they are derived from animal tissue measurements, rather than from human tissue due to the lack of measured data available. The inconsistencies found in this review highlight a gap within the current literature, where conflicting data are being used to model the breast within hyperthermic technology research. Hence, this review provides a thorough evaluation of the available data for the thermophysical properties of healthy and cancerous breast tissues. We also propose a set of reliable values for these thermophysical properties. This compilation will serve as a benchmark data set for modelling breast tissue properties in both hyperthermia and ablation scenarios, while furthering the standardisation efforts within the community.

### 1.1. Breast Tissue Composition

**Figure 1:**
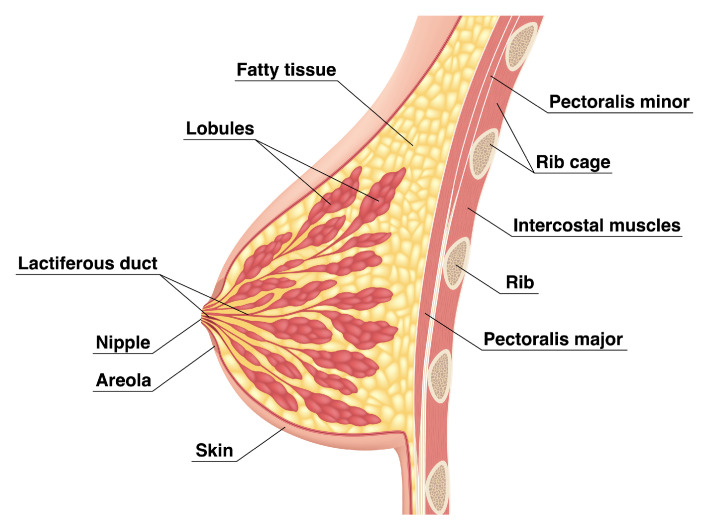
Anatomy of a healthy breast. Image from [14].

Breast is a heterogeneous tissue encased in a layer of skin, consisting of fat and fibroglandular tissue, which is “a mixture of fibrous connective tissue (the stroma) and the functional (or glandular) epithelial cells that line the ducts of the breast (the parenchyma)” [13]. The fatty tissue generally makes up the majority of the breast, acting as the base for the glands (or lobules) and fibrous tissue, as shown in Figure 1. The amount of fatty tissue increases with age and is highly variable between individuals.

**Figure 2:**
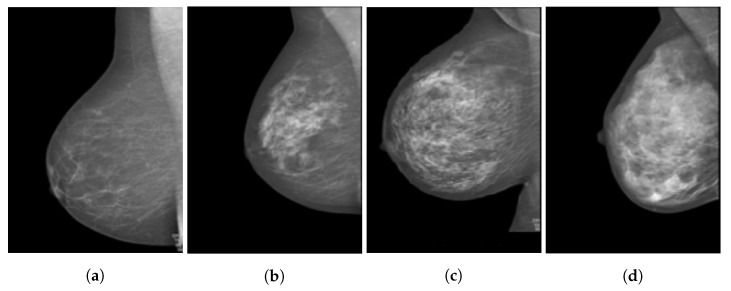
Sagittal cross-section of breast MRI scans showing the breast density variations: (**a**) almost entirely fat, (**b**) scattered fibroglandular tissue, (**c**) heterogeneous fibroglandular tissue, and (**d**) extreme fibroglandular tissue. Images from [16].

The heterogeneity of the tissue makes it difficult to generate standard numerical and physical models, especially since breast density varies from patient to patient. This density is not the numerical ratio of mass to volume, as used in Pennes’ BHE, but rather, a descriptive ratio of fibroglandular to adipose tissues. The more fibroglandular tissue is present, the denser the breast. This breast density can be classified into four categories defined by the American Cancer Society [15]. A comparison of such breast density can be observed in Figure 2, where the four images from (a) to (d) show magnetic resonance imaging (MRI) scans of breasts with increasing fibroglandular tissue composition.

### 1.2. Breast Tumours

Breast cancer can be either invasive/infiltrating (has the potential to spread to other organs) or in situ (noninvasive, does not spread). The most common type of cancer is invasive ductal carcinoma (IDC), which originates in the milk ducts and extends to other parts of the breast over time. In fact, 65–80% of breast cancer patients are diagnosed with IDC [17]. These invasive ductal tumours can sometimes be very hard in texture and are described as scirrhous.

The second most common type of cancer is invasive lobular carcinoma (ILC), which starts in the milk glands (lobules) within the breast and extends through the breast tissue. Approximately 15% of breast cancer cases are ILCs [17]. Tumours originating in the lobules are harder to identify through mammograms than ductal carcinomas [18].

Rarer cancer types include inflammatory breast cancer, an invasive and aggressive cancer, which can be initially misdiagnosed as an infection due to its inflammatory nature. It makes up approximately 1–5% of breast cancer cases [19]. Other rare types of cancer include medullary, colloid (or mucinous), and papillary carcinomas, with each one having an incidence rate of approximately 6%, 2% and less than 1%, respectively [17]. On the other hand, common benign breast diseases include solid fibroadenomas, hamartomas, and fluid-filled cystic lesions.

As with healthy tissue, the properties of tumour tissue also vary depending on the type of tumour. For instance, ductal carcinoma is physically harder than lobular carcinoma. Such variation within tumour tissue makes it impossible to diagnose all cancers using mammography alone. Ultrasound and MRI scans help distinguish solid from cystic masses [20].

### 1.3. Thermal Model of Heat Transfer in Tissue

Pennes’ bioheat transfer equation [10] is the most widely used model for biological heat transfer. The model is frequently implemented in computational simulation software such as CST Studio Suite, Sim4Life, and COMSOL. The bioheat equation (BHE) is written as [21]:

(1) $$\rho c\frac{\partial T}{\partial t}=\nabla \cdot \left(k\nabla T\right)+q_{m}+q_{p}-c_{b}\omega_{t}\left(T-T_{b}\right),$$

where ρ (kg/m${}^{3}$) is the tissue density, *c* (J/kg/K) is its specific heat capacity, *T* (${}^{\circ }C$) is the temperature, *k* (W/m/K) is the tissue thermal conductivity, $q_{m}$ (W/m${}^{3}$) is the metabolic heat generation rate for the tissue, $q_{p}$ (W/m${}^{3}$) is the energy deposition rate into the tissue from a heating source (such as RF, MW, or ultrasound energy), $c_{b}$ (J/kg/K) is the specific heat capacity of blood, $\omega_{t}$ (kg/$m^{3}$/s) is the blood perfusion rate, and $T_{b}$ (${}^{\circ }C$) is the arterial blood temperature, which is often assumed to be equal to the body temperature of 37 ${}^{\circ }C$.

For each biological tissue, the properties ρ, *c*, *k*, $q_{m}$, and $\omega_{t}$ vary and need to be defined. The term $c_{b}\omega_{t}\left(T-T_{b}\right)$ accounts for the removal of heat within the tissue due to the blood perfusion. The blood perfusion coefficient, *B* (W/$m^{3}$/K), can also be used in the BHE, where

(2) $$B=c_{b}\omega_{t}.$$

The blood perfusion rate of tissue, $\omega_{t}$, is presented in different units throughout the literature, and the following relations hold for the most commonly used units:

(3) $$\omega_{t}=\rho_{b}\omega_{t}^{\prime }=\rho_{b}\rho \omega \times \frac{10^{-6}\;m^{3}/\mathrm{mL}}{60\;s/\min},$$

where $\rho_{b}$ (kg/m${}^{3}$) is the density of blood, $\omega_{t}^{\prime }$ is the blood perfusion rate in units of ($m^{3}$/s/$m^{3}$) or (mL/s/mL) or (/s), and ω (mL/min/kg) is the amount of perfused blood in /mL in one minute in 1 kg of tissue (mL/min/kg). The terms *blood flow* and *blood perfusion rate* are used interchangeably within the literature when they mean different things. The blood flow is simply the flow of blood, with units of $m^{3}$/s or L/min. On the other hand, the blood perfusion rate is the rate of blood (kg/s) perfusing through a given tissue volume ($m^{3}$). Hence, within this review, the blood perfusion rates are converted and presented in units of kg/s/$m^{3}$. For the ease of use, Table 1 presents the conversion of units found in the literature to the units used in this paper.

In the case of electromagnetic heating, the terms $q_{m}$ and $q_{p}$ can be expanded as

(4) $$\begin{array}{ccc} q_{m} & = & \rho Q_{m}, \end{array}$$

(5) $$\begin{array}{ccc} q_{p} & = & \rho \times \mathrm{SAR}, \end{array}$$

where $Q_{m}$ (W/kg) is the metabolic heat generated per kg of tissue and SAR (W/kg) is the specific absorption rate.

When considering electromagnetic hyperthermia, current simulation software first computes the electromagnetic solution resulting from the heating device. From the computed fields or specific absorption rate (SAR), the thermal solver uses the BHE to generate the temperature distribution within the tissue. The simulation software calculates this thermal distribution based on the thermophysical properties applied to the model. Some simulation software might have biological tissue properties saved in their material databases, with the thermal properties (amongst other essential properties) already predefined using, for example the IT’IS database as a reference [22].

This review presents an overview of the thermal and physiological properties of human breast tissues as found in the literature. Both measured and estimated data are included for fibroglandular breast tissue, fatty breast tissue, and breast tumour. The data for the review were compiled as described in Section 2. A discussion on the measured and approximated data follows in Section 3. Section 4 presents a compilation of the reliable thermal and physiological properties of healthy and cancerous breast tissues. Finally, Section 5 summarises the review’s findings and contributions.

## 2. Methodology

The thermal and physiological properties that are explored in this review are defined in the BHE: density ρ, specific heat capacity *c*, thermal conductivity *k*, metabolic heat generation rate $q_{m}$, and blood perfusion rate $\omega_{t}$. These properties are required to conduct temperature increase simulations induced by electromagnetic fields or any other heating modality. However, in order to have accurate temperature simulations and compare different heating systems, reliable and standardised tissue properties are critical. This section is a first step towards this goal, where we compiled the thermophysical properties of healthy and tumour breast tissues from the literature.

For this compilation, the blood perfusion rates from both experimental and modelling studies were converted to the SI units of kg/s/$m^{3}$ using Equations (2) and (3) and the conversions in Table 1. Where the quantities ρ, $\rho_{b}$ and $c_{b}$ were provided by the study, these were used to convert the blood perfusion rate of that individual study. When not provided, the quantities as presented in Table 2 were used. The fibroglandular and fatty breast tissue densities ($\rho_{\mathrm{fib}}$ and $\rho_{\mathrm{fat}}$) were obtained from an average of the reliable measurements, as will be discussed in Section 4. Since no reliable measurements of the breast tumour density were found, it was approximated to that of fibroglandular breast tissue. The density of blood was obtained from the IT’IS database [22], which is an average value from four measurement studies [23,24,25,26]. Finally, the specific heat capacity of blood was obtained from an average of two measurement studies [27,28]. While the first study is included as a reference within the database, the other references reported in the database were not considered reliable for this study since they were not measurement studies.

The thermal and physiological properties of human fibroglandular breast tissue, fatty breast tissue, and tumour tissue are presented in Table 3, Table 4, Table 5 and Table 6. The properties were obtained from a review of the current literature, starting from studies that used the thermophysical properties of breast tissue for various modelling or simulation studies. The earliest measurement study identified in this review dates back to 1976. Measurement data were found by tracing back from the referenced sources in these modelling studies and by searching through digital libraries for measurement campaigns. Although tumour tissue properties vary depending on the type of tumour [29], the data for tumour properties are grouped into one section. When specified, the tumour type is listed in the table. However, most simulation studies refer to “tumour” tissue rather than specifying the type of tumour within the model.

The data included in this review are grouped into two categories. The first category consists of the properties obtained directly from measurement campaigns/studies on human breast tissue and breast tumours and are referred to as *Measured* values. These sources are noted in the tables with an asterisk (*). The other category of *Non-Measured* values is a collection of properties found in modelling/simulation studies, where the authors considered approximations or averages of the thermal properties rather than directly measured values. These values were included in the review as they depict the wide range of data considered and the uncertainty in the thermal and physiological properties, especially when measured data are unavailable.

The data reported in Table 3, Table 4, Table 5 and Table 6 are each sorted chronologically, starting from the earliest published study. Table 5 and Table 6 present the properties for human breast tumours, where the former reports *Measured* data and the latter reports *Non-Measured* data. In each table, the first column titled *Reference* gives the first author’s name related to the study. The column titled *Cited by* lists any other studies found that use the same values included in the respective *Reference* column, and were therefore not included as a separate entry in the table. The column *Tissue* notes the type of tissue as specified in the respective *Reference*, while the column titled *Status* gives information on whether measurements were carried out in vivo or in vitro. The column $n_{s}$ provides the number of samples considered in the study, while the column $T_{s}$ gives the temperature at which the samples were measured. These three columns (*Status*, $n_{s}$, and $T_{s}$) are omitted in Table 6 since this table presents breast tumour properties used in modelling studies. Finally, the remaining five columns present the thermal and physiological properties as reported in the *Reference* study.

**Table 3:** The thermal and physiological properties of human fibroglandular breast tissue.

Reference	Cited by	Tissue	Status	$n_{s}$	$T_{s}$	ρ	*c*	*k*	$q_{m}$	$\omega_{t}$
					(${}^{\circ }$C)	(kg/m${}^{3}$)	(J/kg/K)	(W/m/K)	(W/m${}^{3}$)	(kg/s/m${}^{3}$)
Hammerstein (1979) [30] *	[31]	Mammary Gland	in vitro	5	-	1040	-	-	-	-
Gautherie (1980) [32] *	[33,34]	Glandular	in vivo	-	-	-	-	0.370 ± 0.030	-	-
			in vitro	-	-	-	-	0.322 ± 0.009	-	-
		Fibrous	in vivo	-	-	-	-	0.286 ± 0.013	-	-
			in vitro	-	-	-	-	0.255 ± 0.009	-	-
Bowman (1981) [35] *	[23,36]	Atrophic Breast	in vitro	1	37	-	-	0.499 ± 0.004	-	-
		Mammary gland 1 (56.2% Lipid)	in vitro			990	-	-	-	-
Woodard (1986) [31] *	[23,36]	Mammary gland 2 (30.9% Lipid)	in vitro	7	-	1020	-	-	-	-
		Mammary gland 3 (5.6% Lipid)	in vitro			1060	-	-	-	-
Johns (1987) [37] *		Fibrous Breast	in vitro	14	-	1035	-	-	-	-
Erdmann (1990) [24] *	[38]	Breast Glandular	in vitro	5	20	1092 ± 39	-	-	-	-
Ng (2001) [39]	[36,40,41,42,43,44]	Gland ^a^				1080	-	0.480	700	0.571
González (2007) [33]	[40,45]	Breast				920	3000	0.420 ^b^	450	0.189
Bakker (2009) [46]		Breast				1020	2493	0.500	-	0.446
Zastrow (2010) [47]	[48,49]	Fibroglandular and Muscle ^c^				1050	3600	0.500	690	0.745
Jiang (2011) [34]	[41]	Glandular Breast				-	-	0.385	2092	0.571
Chanmugam (2012) [41]	[42,43,50]	Breast Gland				1050	3770	0.480	700	0.630

* *Measured* values. ^a^ Values are from generic muscle tissue and thyroid gland properties obtained from [51]. ^b^ Quoted as effective thermal conductivity (W/m). ^c^ Values are from generic muscle tissue from [52] (study pertaining to head and eye muscles).

**Table 4:** The thermal and physiological properties of human fatty breast tissue.

Reference	Cited by	Tissue	Status	$n_{s}$	$T_{s}$	ρ	*c*	*k*	$q_{m}$	$\omega_{t}$
					(${}^{\circ }$C)	(kg/m${}^{3}$)	(J/kg/K)	(W/m/K)	(W/m${}^{3}$)	(kg/s/m${}^{3}$)
Johnson (1976) [53] *		Normal Breast	in vivo	1	19	-	-	-	-	0.014
Hammerstein (1979) [30] *	[31]	Adipose Tissue	in vitro	8	-	930	-	-	-	-
Gautherie (1980) [32] *	[36,54]	Fatty Breast	in vivo	-	-	-	-	0.171 ± 0.012	-	-
			in vitro	-	-	-	-	0.120 ± 0.008	-	-
Beaney (1984) [55] *	[56]	Normal Breast	in vivo	10	-	-	-	-	-	0.700 ± 0.157
Johns (1987) [37] *	[23,36]	Fat (Breast)	in vitro	14	-	928	-	-	-	-
Robinson (1991) [57] *	[36,58]	Fat (Breast)	in vitro	1	-	934	-	-	-	-
			in vitro	2	37–43	-	2220			
Wilson (1992) [56] *	[59]	Normal Breast	in vivo	17	-	-	-	-	-	0.980 ± 0.245
Hamilton (1998) [60] *	[22]	Breast Fat	in vitro	22	25	-	-	0.209 ± 0.022	-	-
Mankoff (2002) [59] *	[61]	Breast	in vivo	37	-	-	-	-	-	0.978
Ng (2001) [39]	[36,41,42,43,44]	Subcutaneous Fat ^a^				1080	-	0.210	400	0.190
Ekstrand (2005) [54]	[40]	Fat (Breast)				920	3000	0.120	-	-
He (2006) [43]	[42]	Subcutaneous Fat				930	2770	0.220 ^b^	-	-
Converse (2006) [36]	[47,48,49]	Breast ^c^				1069	2279	0.306	350	0.615
Bakker (2009) [46]		Fat (breast)				950	2493	0.240	-	0.416
Jiang (2011) [34]	[41]	Subcutaneous Fat 1 ^d^				-	-	0.246	1180	0.190
		Subcutaneous Fat 2 ^d^						0.385		
Chanmugam (2012) [41]	[50]	Subcutaneous Fat				930	2770	0.210	400	0.210
Singh (2021) [61]		Highly Perfused Fat				920	3000	0.210	400	4.240
		Moderately Perfused Fat				920	3000	0.210	400	8.798

* Measured values. ^a^ Values obtained from generic fat tissue from [51], but they are not exact. ^b^ *k* given in units of (W/m). ^c^ Referenced as “breast tissue”, no distinction between fatty and glandular tissue. However, the properties are cited in other papers as fatty breast tissue. ^d^ *k* was assumed to vary with orientation, so two values of *k* were used in the modelling study.

**Table 5:** Thermal and physiological properties of human breast tumours resulting from experimental measurements.

Reference	Cited by	Tissue	Status	$n_{s}$	$T_{s}$	ρ	*c*	*k*	$q_{m}$	$\omega_{t}$
					(${}^{\circ }$C)	(kg/m${}^{3}$)	(J/kg/K)	(W/m/K)	(W/m${}^{3}$)	(kg/s/m${}^{3}$)
Johnson (1976) [53]		Adenocarcinoma	in vivo	1	24.5	-	-	-	-	1.956
Gautherie (1980) [32]	[33,34,39,40,41,42]	Cancer Tissue	in vivo	-	-	-	-	0.511 ± 0.059	-	-
			in vitro	-	-	-	-	0.280 ± 0.087	-	-
		MucinousCarcinoma ^a^	-	-	-	-	-	0.350	-	-
Bowman (1981) [35]		Scirrhous Carcinoma	in vitro	-	37	-	-	0.397 ± 0.004	-	-
		Mucinous Carcinoma	in vitro	-	37	-	-	0.527 ± 0.041	-	-
Beaney (1984) [55]	[56]	Non-necrotic Tumour	in vivo	10	-	-	-	-	-	3.307 ± 1.662
Valvano (1985) [62]		Breast Adenocarcinoma ^b^	in vitro	3	37	-	-	0.564	-	-
					42			0.584		
Johns (1987) [37]		Infiltrating Duct Carcinoma	in vitro	12	-	1044	-	-	-	-
		Fibroadenoma	in vitro	2	-	1042	-	-	-	-
Robinson (1991) [57]	[36]	Adenocarcinoma ^c^	in vitro	1	37–43	-	3610	-	-	-
		Benign lump (fibrosis)	in vitro	1	37–43	-	3510	-	-	-
Wilson (1992) [56]	[59]	Tumour	in vivo	20	-	-	-	-	-	5.214 ± 2.974
Mankoff (2002) [59]	[61]	Tumour	in vivo	37	-	-	-	-	-	5.968

^a^ The thermal conductivity for mucinous carcinoma is mentioned in the study, but no information is given on whether this value was measured. ^b^ Study gives a linear model of *k* dependence on temperature; the values reported are calculated at 37 °C (body temperature) and 42 °C (hyperthermia). ^c^ From 76-year-old male breast.

**Table 6:** Thermal and physiological properties of human breast tumours resulting from estimations, i.e., *Non-Measured* values.

Reference	Cited by	Tissue	ρ	*c*	*k*	$q_{m}$	$\omega_{t}$
			(kg/m${}^{3}$)	(J/kg/K)	(W/m/K)	(W/m${}^{3}$)	(kg/s/m${}^{3}$)
Ng (2001) [39]	[33,34,36,40,41,42,44]	Tumour (15 mm)	1080	-	0.480	13,600.0	11.429
		Tumour (30 mm)	1080	-	0.480	5790.0	11.429
Ekstrand (2005) [54]	[40,41,42,61]	Tumour (Carcinoma)	1000	3500	0.280	-	-
He (2006) [43]	[41,42]	Tumour	1050	3770	0.480 ^a^	-	-
Converse (2006) [36]		Tumour	1182	3049	0.496	5500.0	1.477
González (2007) [33]	[45]	Cancerous Tissue	920	3000	0.420 ^b^	29,000.0	9.448
Bakker (2009) [46]		Tumour	1000	3770	0.500	-	5.774
Zastrow (2010) [47]	[48]	Tumour ^c^	1050	3600	0.500	690.0	0.745
Jiang (2011) [34]	[41]	Tumour	-	-	0.511	5000.0–65,400.0 ^d^	11.429
Chanmugam (2012) [41]	[50]	Tumour	1050	3852	0.480	5000.0	12.597
Singh (2021) [61]		Highly Perfused Tumour	1080	3500	0.480	10,936.5	22.260
		Moderately Perfused Tumour	1080	3500	0.480	10,936.5	0.530

^a^ Gives thermal conductivity in units of (W/m). ^b^ Quoted as effective thermal conductivity in (W/m). ^c^ Study uses the same properties for tumour and glandular tissue (in Table 3), which were obtained from values of muscle from [52]. ^d^ Range of values depending on diameter of tumour, using an equation from [32].

## 3. Results

In this section, the reviewed *Measured* references are discussed in different subsections, each one pertaining to a different thermal or physiological property. Section 3.6 is dedicated to the remaining *Non-Measured* studies included in the review, with a discussion on how the thermal properties of healthy and cancerous breast tissue were approximated when directly measured values were not available. The discussion closes off with a summary of the data reviewed.

### 3.1. Density

Density (ρ) was the most measured property in this review, with a total of five studies reporting density measurements of healthy breast and tumour tissue. The study by Johns and Yaffe [37] is the only one to report data on the density of each tissue considered: glandular, adipose, and tumour. For tumour, they reported density for both IDC and fibroadenoma (benign breast lumps), which are very similar. Hammerstein et al. [30] reported data on the mammary gland and adipose tissue within the breast. Studies by Woodard and White [31] and Erdmann and Gos [24] only gave data on the density of glandular tissue. Finally, the study by Robinson et al. [57] provided the measured density of fatty breast tissue.

The study by Hammerstein et al. [30] focused on the determination of radiation doses in mammography, but the densities of tissue found in the breast were also calculated, together with the tissue elemental compositions. A distinction between tissue types in the breast was considered for the data collection. Tissue specimens for both glandular and fatty breast tissue were obtained from mastectomy surgeries. The densities were measured by the water displacement method, where the water displaced by a known mass of tissue was measured to find the volume and, hence, the density. Five glandular and eight adipose samples were measured, and an average measurement for each tissue type was obtained, which are presented in Table 3 and Table 4.

Woodard and White [31] reported the values of density (along with water content and elemental composition) of various human tissues, but in particular of glandular breast tissue. The authors distinguished this tissue as being distinct from the adipose tissue found in the breast, but also that the glandular tissue itself varied in lipid content. The measurements on the glandular tissue were carried out on seven samples derived from postmenopausal women. The densities were calculated from the mass proportions of the components of the tissue (water, lipid, protein, and ash) and the mass densities of each of the components. Since more than five sets of measured data were obtained, the authors categorised the tissue properties into three groups based on the standard deviation of their data. One group contained the averaged measured values *M*, while the other two groups considered the standard deviations σ for all reported properties and quantities. The three categories considered are therefore mammary gland 1 (M−σ), mammary gland 2 (*M*), and mammary gland 3 (M+σ).

The study by Johns and Yaffe [37] was not directly related to obtaining thermal properties of breast tissue, but rather attenuation measurements when being irradiated with X-rays. The densities of fibrous and fat tissue within the breast along with those of IDC and fibroadenoma are here summarised. Healthy fat and fibrous tissue samples were obtained from 14 patients either undergoing surgery or autopsies. The IDC density was measured from samples obtained from 12 mastectomy or lumpectomy patients, while the fibroadenoma measurements were carried out on samples from two patients who underwent a lumpectomy. For most of the specimens, the tissue was stored frozen for a period of time and then cut into blocks of the required dimensions for the attenuation measurements. Once thawed, attenuation measurements were carried out and the tissue was then refrozen. The density was determined once the tissue had been thawed and excised once again, through measurements of buoyancy in phosphate-buffered saline. The processes of freezing and thawing the tissue samples twice before calculating the density most likely led to some hydration loss and, therefore, the loss of some mass, altering the final density measurement. At least five buoyancy measurements were taken on each tissue specimen and averaged. The overall error for this measurement technique was 7 kg/$m^{3}$.

The study by Erdmann and Gos [24] focused on the density of various trunk tissues, which included measurements of glandular breast tissue. Tissue samples were obtained from ten cadavers of both sexes, but the authors did not disclose how many were male or female, nor did they report the number of samples collected per tissue. The tissues obtained from autopsies were stored at 4 ${}^{\circ }C$. Around three to six hours later, the tissues were placed at room temperature, and after at least one hour, the densities were measured. The weight of each sample was obtained using a laboratory scale of accuracy ±0.001 g, while the volume was obtained by placing the sample in a 25 ${\text{cm}}^{3}$ pycnometer filled with water. Seven measurements of mass and volume were obtained for each sample, where the lowest and highest calculated densities were omitted from the results to obtain an average of the remaining five calculations. A relative error of less than 0.5% was reported for the averaged values.

Robinson et al. [57] investigated tissue-mimicking phantoms for hyperthermia applications, but also conducted thermal measurements of breast fat and breast tumour tissue. Tissues in this study were obtained from biopsies, and the measurements were conducted on one sample for each tissue type. The sample for breast fat was attached to a fibrosis from a 63-year-old female, which was also measured. The breast adenocarcinoma sample was obtained from a 76-year-old male. The samples were stored at 4 ${}^{\circ }C$ in airtight containers and measured at most 24 h after excision. The tissue density of the breast fat was found using Archimedes’ method of water displacement [63].

### 3.2. Specific Heat Capacity

The only measurement data found for the specific heat capacity (*c*) of breast tissue and tumour are those of Robinson et al. [57]. The authors used the Perkin Elmer DSC2 (PerkinElmer, Waltham, MA, USA) differential scanning calorimeter to carry out measurements. The specific heat capacities of two samples of fatty breast tissue were measured over the range of 25–50 ${}^{\circ }C$, but the value reported in the study is an average of the specific heat capacity considered over the range 37–40 ${}^{\circ }C$. This temperature range was considered since it is the most commonly used in hyperthermia therapy, according to the authors. The study also notes a physical phase change in fat within the temperature range of 28–36 ${}^{\circ }C$. The specific heat capacity of fibrosis and adenocarcinoma was also measured over the same temperature range. However, these specific heat capacities are reported in units W/m/K, which correspond to units of thermal conductivity, and not in units of J/g/K, as was the case for other specific heat capacities reported in the paper. Since the authors report these values in the same paragraph as the specific heat capacities of other tissues and from the order of magnitude of these quantities compared to those of thermal conductivity, we concluded that these values are indeed specific heat capacities reported in the wrong units. These are presented in J/kg/K in Table 5.

The IT’IS tissue properties database considers an approximation equation for the calculation of the specific heat capacity of breast glandular tissue. This equation uses the specific heat capacity and mass fraction of each tissue component, as described by [64] (cited in [23]). The components are water, fat, and protein, and the specific heat capacity can be approximated using

(6) $$c=\sum_{n=1}^{3}w_{n}c_{n},$$

where $w_{n}$ is the mass fraction and $c_{n}$ is the specific heat capacity of the $n^{\mathrm{th}}$ component. Riedel [65,66] (cited in [23]) assumed that the proportions of fat and protein are equal, so that the equation can be simplified to

(7) c=1670+25.1W,

where *W* (%) is the percentage water content of the tissue. This empirical equation was derived after measuring various meat and fish tissues and is used to calculate the specific heat capacity of glandular breast tissue in the IT’IS database [22], where the water content W= 51.4% measured by Woodard and White [31] was used for this approximation.

### 3.3. Thermal Conductivity

There are four studies that provide measurements for the thermal conductivity (*k*) of breast tissue and tumour. Gautherie [32] performed a large-scale study on the thermal conductivity of healthy and tumour tissues, giving values for in vivo and in vitro measurements. Bowman [35] measured the thermal conductivity of breast tissue and two types of breast cancers (scirrhous and mucinous), but provided no indication of the adipose content of the “normal atrophic tissue”. Valvano et al. [62] devised a method for measuring the thermal conductivity using a self-heated thermistor. They obtained linear regression models for the thermal conductivity of various tissues including breast adenocarcinoma. Finally, Hamilton’s Ph.D. thesis [60] reports thermal conductivity measurements of fatty breast tissue using the method devised in [62].

Gautherie [32] conducted in vivo and in vitro measurements of the thermal conductivity of breast tissues and breast tumours. The measurements were carried out using fine-needle thermoelectric probes (0.8 mm in diameter) and a fluvograph on 147 patients. The methods and apparatus were described in a previous paper by the author and colleagues [67], but a version of this paper could not be found. The in vivo measurements were carried out under local anaesthesia, where a probe was inserted into the tumour within the cancerous breast and another probe was inserted simultaneously into the contralateral healthy breast at approximately the same location. Measurements were taken starting from a maximum depth of 6.5 cm (the length of the needle) and while extracting the needle at intervals of 5 mm. The in vitro measurements on healthy tissue were taken on postoperative samples from mastectomies or benign growth excisions. The healthy breast tissue was classified into either glandular, fibrous, or fat tissue, depending on the tissue appearance.

The author notes that the differences in the thermal conductivity for in vivo and in vitro samples are due to heat removal from the blood perfusion of in vivo tissue. In healthy tissue, the thermal conductivity is higher in the in vivo scenario by approximately 0.05 W/m/K. This thermal conductivity increase for in vivo measurements (compared to in vitro values) amounts to a 14.9%, 12.2%, 42.5%, and 82.5% increase for the glandular, fibrous, fatty, and tumour tissue, respectively, as considered in this study. The difference between these values is a function of the blood perfusion levels in the tissue. In fact, tumours require significant perfusion levels to feed their high metabolic demands, which is in line with the significant thermal conductivity changes in perfused vs. unperfused (in vitro) tumour tissue. On the other hand, fibrous tissue is among the least-perfused tissues, which is also represented in the smallest thermal conductivity increase due to perfusion among the analysed tissues, assuming that the connective heat flow is isotropic, as established in mathematical models [68]. A difference in the blood perfusion of 1500 mL/min/kg gives a change of 0.05 W/m/K in the thermal conductivity.

While there is a notable difference in the thermal conductivity for in vivo tumour tissue compared to healthy tissue, the measured thermal conductivity for excised tumour compared to excised healthy tissue is not so distinct. An indication of this is the standard deviation, which is presented in Table 5. The measured thermal conductivity of in vitro tumour tissue ranged from approximately 0.10 to 0.45 W/m/K within the study. This clearly shows the thermal conductivity dependence on perfusion status, hinting that the measurement of the other physical properties is also dependent on blood perfusion.

The study carried out by Bowman [35] presents measured values of the thermal conductivities of various tissues, specifically breast tissue and breast tumours. The fat content of the healthy tissue was not distinguished, and there was no mention of the samples being fibroglandular or fatty in nature. The measurement for breast tissue was obtained from one in vitro sample at 37 ${}^{\circ }C$. The study also includes the thermal conductivity of scirrhous and mucinous (colloid) breast carcinomas, together with tumours in other organs/sites. The thermal conductivities for all tumours measured in this study (not just breast tumours) were all higher than those of the adjacent healthy tissues considered, except for the scirrhous carcinoma in the breast and colonic carcinoma.

The measurement method for these results is not discussed in the paper. However, the study does mention the thermal diffusion probe, which is capable of measuring the thermal conductivity together with the temperature, thermal diffusivity, and perfusion of tissues. There is no specific mention that the thermal diffusion probe was used to measure the thermal properties of human tissue, but the author describes the thermal diffusion probe method in relation to other canine and rat thermal conductivity measurements against time, within the same paper.

The study by Valvano et al. [62] tackles the temperature dependence of the thermal conductivity. The authors describe a self-heated thermistor probe method of measuring the thermal conductivity simultaneously with the thermal diffusivity. Through this method, they obtained linear regression models for these properties of various animal and human tissues. Of interest to this review are the data on the thermal conductivity of breast adenocarcinoma.

Three samples of adenocarcinoma were obtained from biopsies, on which a total of 100 measurements were conducted within 24 h from excision. The thermal conductivity was measured at temperatures of 3, 10, 17, 23, 30, 37, and 45 ${}^{\circ }C$. From these measurements, a linear model of the thermal conductivity as a function of temperature was obtained:

(8) $$k=k_{0}+k_{1}T,$$

where *k* is the thermal conductivity (W/m/K) and *T* (${}^{\circ }C$) is the temperature. $k_{0}$ and $k_{1}$ are the coefficients obtained through the linear regression fit and are equal to 0.4194 W/m/K and 0.003911 W/m/K/${}^{\circ }C$, respectively. The correlation coefficient for the linear regression was found to be 0.60. Temperatures *T* of 37 ${}^{\circ }C$ and 42 ${}^{\circ }C$ were considered to obtain the thermal conductivity at a baseline and mid-hyperthermic temperatures, which are reported in Table 5.

Measurements were conducted using a method devised by the authors. First, a spherical thermistor probe is inserted into the tissue, and a baseline temperature $T_{s}$ is measured using the thermistor in passive mode. Then, a voltage V(t) is applied such that the temperature at the probe increases by around 4 ${}^{\circ }C$. This new temperature $T_{h}$ is kept constant for 20 s by varying the voltage and, hence, the power Q(t). The power at a time *t* during heating is calculated through the equation:

(9) $$Q\left(t\right)=\frac{V{\left(t\right)}^{2}}{R},$$

where *R* is the resistance of the feedback circuit used to heat the thermistor. The voltage is measured at intervals of 0.5 s, and hence, Q(t) can be evaluated using Equation (9). This power is characterised as

(10) Q(t)=P+St−12,

where *P* and *S* are the steady-state term and the transient term, respectively. These terms can be found from a plot of the power Q(t) against t−12 in the interval where the elevated temperature $T_{h}$ is kept constant. The thermal conductivity *k* can then be found through the equation:

(11) k=1A(ΔTP)+B,

where $\Delta T=T_{h}-T_{s}$ and *A* and *B* are constants obtained through the calibration of the probe on solutions of well-known thermal conductivities.

In Hamilton’s thesis [60], measurements of the thermal conductivity (and water content) of breast fat samples were obtained. A total of 22 samples were obtained from three patients. Two of these samples were obtained through surgery on two patients, whilst the other 20 samples were obtained from a postmortem autopsy on one patient. Ten measurements were performed on each sample for a total of 220 measurements. The average thermal conductivity for human breast fat was found to be 0.209 W/m/K, with a minimum measurement of 0.171 W/m/K and a maximum of 0.253 W/m/K. The measurements were conducted using the same method in Valvano et al. [62].

As with the specific heat capacity, the thermal conductivity, *k*, of tissues can also be approximated using the water content. The equation by Cooper and Trezek [64] cited by Duck [23] gives such a relation for the thermal conductivity of tissue:

(12) k=0.0502+0.00577W,

where *W* (%) is the water content of the tissue. This relation is also referred to in the IT’IS database, where the thermal conductivity of glandular breast tissue is calculated using the average water content reported in [31] of 51.4%.

Another equation for estimating the thermal conductivity is given by Poppendiek et al. [25] in Duck [23], where the equation uses the properties of the components of the tissue. The three components considered are fat, protein, and water, and the thermal conductivity is estimated by:

(13) $$k=\rho \sum_{n=1}^{3}\frac{w_{n}k_{n}}{\rho_{n}},$$

where $w_{n}$ is the mass fraction, $k_{n}$ is the thermal conductivity, $\rho_{n}$ is the density of the nth component of the tissue, and ρ is the density of the tissue as a whole.

#### Note on Effective Thermal Conductivity

The aforementioned measurement studies involve both in vivo and in vitro measurements of the thermal conductivity. However, an important distinction should be made for these two scenarios. The in vivo measurements by Gautherie [32] are referred to as “effective” thermal conductivities, where the blood perfusion of the tumour influences these measurements. In in vivo scenarios, heat is transported by convection through the capillary vessels while using the thermoelectric probe. This heat removal through blood perfusion allows for an increase in energy deposition within the same volume of tissue when conducting in vivo measurements, compared to the allowed energy deposition in in vitro measurements. In fact, the mean in vitro measurements presented by Gautherie [32] are significantly lower than the reported in vivo values. On in vitro samples, the thermal conductivity is measured according to Fourier’s law, where the heat transport due to convection within the blood vessels does not influence the measurements. These in vitro measurements are referred to as “intrinsic” thermal conductivities by Bowman [35].

### 3.4. Metabolic Heat Generation Rate

There are no *Measured* sources for the metabolic heat generation rate ($q_{m}$) of healthy breast tissue. However, the study by Gautherie [32] explores the dependency of the metabolic heat generation on the volume doubling time of breast tumours, demonstrating that they are inversely proportional through a scatter plot. The shorter the tumour doubling time is, the more heat is generated by the tumour. From these data, Ng et al. [39] developed the following relation:

(14) $$q_{m}\tau =C,$$

where $q_{m}$ (W/m${}^{3}$) is the metabolic heat production, τ (days) is the tumour volume doubling time, and *C* is a constant equal to $3.27\;\times \;10^{6}$ W day / m${}^{3}$. Ng et al. also developed a relation between the volume doubling time τ to the tumour diameter *D* (m):

(15) $$D=\exp\left[0.002134\left(\tau -50\right)\right]\times 10^{-2},$$

These two relations are not specifically mentioned in Gautherie’s 1980 paper [32], but Ng et al. cited an earlier study by Gautherie [69] as the source for these equations. Through these equations, the doubling time of the tumour, and hence the metabolic heat generation rate, can be obtained if the tumour diameter is known. In fact, some studies [34,40,61] use these equations to obtain the tumour metabolic heat generation rate values for the tumour diameter considered in their models/simulations.

The metabolic heat term in the BHE is sometimes omitted from simulations to simplify calculations. The term has a small, almost negligible effect when compared to other processes in the model such as the external heating from, e.g., microwaves, and the blood perfusion in tissues [70].

### 3.5. Blood Perfusion

The blood perfusion term in the BHE is a major heat sink that counteracts the external heating during hyperthermia treatments. This review found four studies, Johnson [53], Beaney [55], Wilson et al. [56], and Mankoff et al. [59], that measured the blood perfusion rates of healthy and cancerous breast tissue. In each case, the fibroglandular or fat content in the healthy tissue was not specified, but the measurements on healthy breast tissue were listed as fatty breast tissue properties in Table 4. Johnson [53] conducted measurements using a thermal approach, while the other three studies were conducted using positron emission tomography (PET) imaging on breast cancer patients. Each study gives the measured blood perfusion rates in different units of measurement, which have been converted to units of kg/s/$m^{3}$ for comparison, as explained in Section 2.

Johnson [53] conducted blood perfusion measurements on in vivo patients using a thermodynamic approach to compare the blood perfusion of tumours before and after radiotherapy. This early study involved blood perfusion measurements on various human tumours, including measurements of healthy and cancerous breast tissue for two patients. The apparatus consisted of a copper heat sink placed over the tissue, which was kept at various temperatures ranging from hypothermic to hyperthermic through a water circulator and measured using a thermistor. A heat flow sensor was placed between the copper plate and the patient’s skin. The heat flux, surface skin temperature, and patient’s internal temperature were monitored until thermal equilibrium was reached, after which these three quantities were recorded over 10 min.

The paper reports blood perfusion measurements for two patients, but only the data for one patient could be extracted. The measurement data within the paper are given through plots of blood perfusion over a number of measurement days before and after radiotherapy. The blood perfusion in the paper is defined as $Wb=\frac{Q}{(t_{1}-t_{2})K}$, where *Q* is the heat flux, $t_{1}$ is the patient’s oral temperature, $t_{2}$ is the skin surface temperature, and *K* is undefined. The heat flux units vary within the paper itself, initially stating that it was recorded in μV, but the heat flux disk was calibrated in units of bthu/in^2^/s/mV. Furthermore, the plots of blood perfusion are only labelled in symbol form as $\frac{Qa}{t_{1}-t_{2}}$, where *a* is undefined. These conflicting units of measurement make it impossible to extract data from these plots with any certainty. The data reported in Table 4 and Table 5 were obtained from subsequent plots within the paper where the measurements were converted to units of mL/g/min using an estimation program referenced in the study, but unavailable online. The data from the plots were extracted using the GetData Graph Digitizer [71] and converted to SI units using the conversion presented in Table 1 and the appropriate quantities given in Table 2. The data extracted from the graph digitiser resulted in blood perfusion rates of $8.32\;\times \;10^{-4}$ mL/g/min and 0.105 mL/g/min for healthy and cancerous breast tissue, respectively, for measurements conducted before radiotherapy. This study showed a clear increase in the blood perfusion through breast tumour tissue compared to healthy breast tissue. The perfusion values were converted to the SI units used in this review through the appropriate conversion in Table 1 and the densities of fatty breast and tumour tissue presented in Table 2, which resulted in 0.014 kg/s/$m^{3}$ and 1.956 kg/s/$m^{3}$ for healthy and cancerous breast tissue, respectively.

Beaney [55] conducted in vivo blood perfusion measurements using PET scans. Imaging was performed on ten patients whose ages ranged from 52 to 74 years using an ECAT II PET scanner, according to an earlier study, which reported more detail on the measurements, but with information on nine out of the ten patients [72]. For each patient, the blood perfusion was measured for both tumour and normal breast tissue. The mean blood perfusion of tumour tissue was 18.78 ± 9.67 mL/100 mL/min (or 3.307 ± 1.662 kg/s/$m^{3}$). For healthy tissue, a mean blood perfusion of 4.00 ± 0.90 mL/ 100 mL/min (or 0.700 ± 0.157 kg/s/$m^{3}$) was obtained. The blood perfusion in healthy tissues was obtained by measuring perfusion in the healthy contralateral breast. Although the average tumour blood perfusion was higher than that of healthy breast tissue, the tumour blood perfusion measurements ranged from 7.76 to 31.40 mL/100 mL/min (or 1.358–5.495 kg/s/$m^{3}$) according to the earlier published paper [72]. The lower value for the blood perfusion of tumour tissue was still higher than the highest measured healthy breast blood perfusion, which ranged from 2.74 to 5.66 mL/100 mL/min (or 0.479–0.990 kg/s/$m^{3}$).

Wilson et al. [56] performed blood perfusion measurements on breast tumours and healthy tissue. The measurements were conducted on a total of 20 patients where the ages varied from 35 to 77. Each patient was diagnosed with IDC, except for a male patient, who was diagnosed with lobular carcinoma of the breast. The patients had lesions of different stages and were undergoing either no treatment, hormone therapy, chemotherapy, or radiotherapy at the time of study.

The blood perfusion measurements were conducted using an ECAT 931-08/12 PET scanner. The blood perfusion of tumour tissue was significantly larger than that of healthy tissue, as can be seen from the results presented in Table 4 and Table 5. The average blood perfusion for tumour tissue was 29.8 ± 17.0 mL/min/dL (or 5.214 ± 1.662 kg/s/$m^{3}$), whilst that of healthy tissue was 5.6 ± 1.4 mL/min/dL (or 0.980 ± 0.245 kg/s/$m^{3}$). The measured blood perfusion of the tumour tissue ranged from 11.3 to 76.8 mL/min/dL (or 1.977 to 13.437 kg/s/$m^{3}$), and no association could be made between the perfusion and tumour size. However, it was noted that three of the patients who developed rapid progressive and metastatic disease and died within three months of the study had the highest measured tumour blood perfusion.

Mankoff et al. [59] also measured the blood perfusion rate of healthy and cancerous breast tissue. The measurements using PET imaging were carried out on 37 patients with newly diagnosed locally advanced breast cancer. ^18^F−FDG and ^15^O−water tracers were used in the imaging of tumours ranging from 1.9 to 11.0 cm. The study gives an average blood perfusion of 60 mL/min/kg (or 0.978 kg/s/$m^{3}$) for healthy tissue and 320 mL/min/kg (or 5.968 kg/s/$m^{3}$) for tumour tissue. Although there is a notable difference between these two values, the blood perfusion of tumour tissue varies greatly and the measurements within the study overlap those of healthy tissue.

Despite the different values between these three PET measurement studies, they all share a similar blood perfusion ratio between tumours and healthy breast tissue: 4.7 for Beaney [55] and 5.3 for both Wilson et al. [56] and Mankoff et al. [59].

### 3.6. Non-Measured Studies

In this section, we summarise the modelling studies that used *Non-Measured* values of the thermal and physiological properties of breast tissues in their simulations. In these *references*, the authors estimated the thermophysical properties due to lack or difficult access to experimental measured data. Instead, they used approximations based on tissues with a similar tissue composition. Furthermore, most authors quote values from other sources, which, in turn, retrieve their properties from another source. Following this trail of references, some properties were in fact from measurement studies, but other values were misquoted or taken from sources pertaining to tissues that were not breast. As for breast tumour tissues, when measured values were not available, the thermal properties were sometimes assumed to be the same as those of glandular tissue or from generic tumour measurements from other sites.

The study by Ng et al. [39] focused on the numerical modelling of a female breast with a tumour. The model consisted of a three-dimensional hemisphere with an outer skin layer with a subcutaneous fat layer. The majority of the breast was modelled as glandular tissue, with a layer of muscle to mimic the chest wall and the tumour inserted in the glandular tissue. The parameters given for the tissue used were density, thermal conductivity, metabolic heat generation rate, and blood perfusion coefficient. The densities were kept the same for each tissue at 1080 kg/$m^{3}$. Most of the thermal properties were obtained from approximations from Werner et al. [51]. The thermal conductivity of breast glandular tissue was possibly obtained from that of the thyroid gland. While this was not explicitly stated and there were multiple tissues with the same thermal conductivity in Werner et al., the thyroid gland is the closest possible tissue reported. Ng et al. also used the same thermal conductivity for tumour tissue as that of glandular. The remaining properties of glandular tissue were approximated from those of muscle tissue, whilst the data for the subcutaneous fat were obtained from generic fat measurements.

The referenced study by Werner et al. reports values for the basal metabolic heat production in units of W while giving the respective volumes for the tissue. The metabolic heat production of glandular tissue can be calculated from these values of muscle, with a basal metabolic heat production of 18.93 W and volume of 27,338 ${\text{cm}}^{3}$. For fat, a basal metabolic heat production of 3.74 W for a volume of 10,153 ${\text{cm}}^{3}$. was listed. Dividing the basal metabolic heat production by the volume gives approximate values for the metabolic heat production of 700 W/$m^{3}$ for muscle and 400 W/$m^{3}$ for fat. These are the values noted in [39] for glandular tissue and fat, respectively.

For the blood perfusion coefficient of glandular and fat tissue, the authors also considered values from [51], possibly obtained from the values for muscle and fat (the authors did not specify). These quantities were then converted using Equation (2) and the specific heat capacity of blood in Table 2 to obtain the quoted values in units of kg/s/$m^{3}$.

Tumour tissue properties, specifically the metabolic heat generation rate and blood perfusion coefficient, were obtained from a study by Gautherie [32]. The metabolic heat production for various tumour sizes can be calculated from the tumour diameter, as discussed in Section 3.4, while the blood perfusion coefficient was estimated from observations by Gautherie, [32], Priebe [68], and Vaupel et al. [73].

The study by Ekstrand et al. [54] considered a basic model of a human breast with a tumour to determine whether the tumour is preferentially heated during ablation. The breast model consisted of a sphere with fat properties with an embedded ellipsoidal tumour. The densities and specific heat capacities were gathered from sources that report the properties for generic fat tissue and tumour (not in the breast) [51,74]. However, the thermal conductivities were obtained from a 1975 study by Gautherie et al. [69]. A copy of this earlier study by Gautherie could not be found, but the quoted values are the same as the in vitro measurements presented in the Gautherie’s 1980 study [32], where the experimental data are included and discussed in this review.

He et al. [43] considered a model of mostly glandular tissue with an embedded tumour and a layer of subcutaneous fat encasing the glandular tissue. According to the citations in the study, the thermophysical properties were obtained from [39,75,76,77]. However, the values given for fat do not match other values given in the sources. Possibly, the authors obtained an average from some or all sources. The thermophysical properties of tumour tissue from this study are included in this review in Table 6. However, the tumour properties in this study are identical to the ones given for glandular tissue, which, in turn, are directly quoted in Chanmugam et al. [41]. Hence, they are not listed in Table 3. The study by Chanmugam et al. is included in the tables given in Section 3 as more information about the thermal properties of glandular tissue is included and will be discussed in this current section.

The study by Converse et al. [36] considered an anatomically realistic breast model, where the dielectric properties of the tissue varied depending on the fat content of the breast. However, the same approach was not adopted for the thermal properties, and the healthy breast tissues were given the same thermophysical properties regardless of fat content. Since the thermal properties of breast tissue in this study were used as the properties of fatty breast tissues in later studies [47,48,49], the properties from Converse et al. are included in Table 4 representing fatty breast tissues and not in the fibroglandular breast properties table. The authors listed 18 sources as the references for the thermal properties. As such, it is not possible to distinguish the exact sources for each individual tissue, and most likely, an average was obtained. The 18 sources include studies on the thermal properties of breast (with no distinction between fatty or fibroglandular tissue), muscle, fat, skin, and tumour.

The model used in the study by González [33] consisted of a hemisphere of breast tissue with an embedded spherical tumour. A layer of muscle below the breast hemisphere was added to mimic the chest wall. The density and heat capacity were the same for both normal breast tissue and tumour. The study considered the breast hemisphere of radius 9 cm so that the study mimicked an example presented by Gautherie et al. [32]. The thermal conductivity (used for both glandular and tumour tissue) and the metabolic heat generation rate of the tumour were the exact values as calculated in the Gautherie study for this specific example. There is no reference given for the density, specific heat capacity, and blood perfusion rates for healthy and cancerous tissue.

Bakker et al. [46] investigated ultrasound hyperthermia applied to the breast, where they assigned thermal properties to breast, fat, and tumour tissues. Simulations were carried out on a breast anatomical model based on an MRI scan. This model depicted a breast with a background of fat and breast tissue within that fat. There was no indication that this breast tissue is glandular, but they referred to the tissue as breast tissue. The only traceable values in this study are the thermal conductivity of breast tissue and the density and thermal conductivity of fat tissue. The thermal conductivity of breast tissue can be traced back to the measured value of Bowman [35]. The density and thermal conductivity of fat can be traced back to that of generic adipose tissue from Woodard and White [31] and Lang et al. [78], respectively. It is worth noting that although the reference trail leads to [78], the value for the thermal conductivity in the primary source is that of 0.21 W/m/K, whilst that given in Bakker et al. is 0.24 W/m/K.

The density of breast tissue and the specific heat capacity and thermal conductivity of tumour tissue can be traced back to Sohrab et al. [79]. These values were cited from a Ph.D. thesis not available digitally. The remaining properties, i.e., the specific heat capacity of fibroglandular breast and breast fat, the tumour density, and the blood perfusion rates for the three tissues considered, do not match the quantities given in Sohrab et al. Since this was the only reference given in Bakker et al., the sources for these properties could not be traced. However, for comparison in this review, the blood perfusion rates were converted into the SI units using the conversion in Table 1 together with the tissue densities provided by the authors and the blood density in Table 2.

Zastrow et al. [47] present a computational study of microwave hyperthermia for breast cancer, which included patient-specific breast models. The four models considered range from mostly fatty tissue to denser models of mostly glandular tissue, which are based on MRIs such as those in Figure 2. The thermal properties of fibroglandular tissue were assumed to be the same as those for muscle tissue and were taken from [52], a study that considered muscle tissue found in the head and eye. Throughout the study, the tumour was embedded in the fibroglandular tissue, and no dielectric or thermal contrast was considered. The thermal properties of fatty breast tissue and skin were quoted directly from [36] and are therefore not repeated in Table 4.

Jiang et al. [34] considered a hemispherical model made of skin, subcutaneous fat, and two glandular tissues called *core gland* and *sub gland*. However, there was no distinction made between the thermal properties of the different types of glandular tissue, with the tissues being listed as glandular, fat, skin, and tumour. The authors referenced the thermal conductivity, metabolic heat generation rate, and blood perfusion coefficient to those from in vivo measurements by Gautherie [32], but the only specific values presented in [32] relate to the thermal conductivity of breast and tumour tissues. Furthermore, the value given for the in vivo measurements of the thermal conductivity of glandular breast tissue in Gautherie’s work is 0.37 W/m/K, and not 0.385 W/m/K, as reported by Jiang et al. The thermal conductivity of the fat tissue within this study was assumed to be anisotropic, where the values varied with the orientation of the tissue with respect to the axes considered. As for the metabolic heat generation rate and the blood perfusion coefficient in Jiang et al., there is also no specific mention of these quantities in Gautherie’s 1980 work [32]. However, Jiang et al. considered the same calculations as Ng et al. [39], where the metabolic heat was determined from tumour diameter. Although not directly stated, it seems the blood perfusion coefficients for glandular tissue, fat, and tumour were obtained from this study by Ng et al. The blood perfusion coefficients were converted to the blood perfusion rates using Equation (2) and the specific heat capacity of blood presented in Table 2.

Chanmugam et al. [41] considered a model where the large part of the breast was made of glandular tissue, with a subcutaneous fat layer and a muscle layer below the gland in lieu of the chest wall. The tumour was embedded in the glandular tissue. For both glandular and fat tissues, the thermal conductivity and metabolic heat generation rate were cited from Ng et al. [39]; the densities and specific heat capacities can also be traced back to Ng et al. However, in Chanmugam et al., these latter values are slightly different than the ones listed in the cited study. The blood perfusion rate values reported are also similar, but not exactly the same as those in Ng et al. For tumour tissue, the density and thermal conductivity can be traced back to Ng et al., whereas the specific heat capacity was quoted from that of a skin lesion in Çetingül et al. [80]. The metabolic heat generation rate was retrieved from the minimum value provided by Jiang et al. [34], who, in turn, used the tumour diameter and equations reported in Ng et al. [39] to obtain the metabolic heat generation rate, as discussed in Section 3.4.

The study by Singh et al. [61] considered a computational breast model for ablation research. The breast model was made of mostly fatty breast tissue, with a tumour embedded within the fat. The density, specific heat capacity, thermal conductivity, and metabolic heat generation rate of both fat and tumour tissues were obtained from sources already listed and discussed in this review [39,40,54,69]. The tumour metabolic heat generation rate was obtained from the equations given in Ng et al. [39] while considering a tumour diameter of 1.7 cm. The blood perfusion rates for both fat and tumour given in this study have not been discussed in this review yet. Two levels of perfusion were assigned to both fat and tumour tissue: highly perfused tissue and moderately perfused tissue. The values for highly perfused healthy and tumour tissue were obtained from Mankoff et al. [59], whilst those for moderately perfused healthy and tumour tissue were obtained from Fujita et al. [81]. In the former study, the blood perfusion measurements were carried out using PET imaging of cancerous breast tissue (discussed in Section 3.5), while Fujita et al. [81] measured the blood perfusion of the healthy abdomen and tumour within the abdomen (the specific tissue was not specified). The values presented in Table 4 and Table 5 are converted to the SI units from the original study’s units of /s by multiplying them with the tissue densities as specified by the study.

However, the blood perfusion values used by Singh et al. do not make physical sense. Firstly, moderately perfused breast fat is assigned a higher perfusion rate than highly perfused breast fat. Secondly, the tumour perfusion values for moderately and highly perfused tumours are 0.530 and 22.260 kg/s/$m^{3}$, respectively, which are outside the reported tumour values by Mankoff et al. (1.89–16.19 kg/s/$m^{3}$). The same holds for breast tissues, where Singh et al. reported values of 8.80 and 4.24 kg/s/$m^{3}$ for moderately and highly perfused tissue, respectively, while the measured range by Mankoff et al. is 0.46–2.46 kg/s/$m^{3}$.

### 3.7. Summary

Density is the easiest property to measure, leading to more measurements being available on breast density. The distinction between glandular and fatty tissue within the breast was made in each study. However, some measurement conditions might have resulted in undesirable errors. For instance, in the study by Johns and Yaffe [37], the tissue samples underwent two freezing and thawing processes before the densities were measured. Similarly, in Erdmann and Gos [24], the samples were kept at 4 ${}^{\circ }C$ before bringing them up to room temperature and then measuring the density. There were no indications on how long the samples had been left out before being measured. In some instances, the samples were even obtained from autopsies [24,37]. These factors all contribute to alterations in the water content of the samples and, hence, introduce an unpredictable error within the measured density. Different methodologies were used to measure the densities. The water displacement method was used in [30,57]. The buoyancy of the samples was used to calculate the densities in [37], whereas a pycnometer was used in [24]. Finally, the density of the components of the tissue, together with the mass proportions of the components within the tissue were used in [31].

Only one measured source was found for the specific heat capacity, which is Robinson et al. [57]. The authors presented measurements of breast fat, adenocarcinoma, and benign fibrosis. Only two breast fat samples and one sample of adenocarcinoma and fibrosis were included in this study.

Four studies measured the thermal conductivity of breast tissue and tumour. The most thorough is the one by Gautherie [32], where the author performed measurements on a total of 147 patients in the measurement campaign. The measurements were conducted both in vivo and in vitro, using a thermoelectric probe, while also considering the tissue composition of glandular, fibrous, fat, or tumour tissue. Bowman [35] also measured the thermal conductivity of healthy breast tissue, scirrhous carcinoma, and mucinous carcinoma. However, the measurement method was not described and the fat content in the sample of breast tissue was not specified. Valvano et al. [62] describes a method for measuring the thermal conductivity and thermal diffusivity of tissues simultaneously using a self-heating thermistor probe. The method was used to obtain a linear regression model of the thermal conductivity of adenocarcinoma samples as a function of temperature. Three samples were obtained from biopsies, and a total of 100 measurements were conducted not more than 24 h after excision. Finally, Hamilton [60] measured the thermal conductivity of breast fat in his thesis from 22 samples. Although this was quite a large sample size, 20 of these samples were obtained from an autopsy of one patient. The author used the same self-heated thermistor probe method as Valvano et al. [62].

Although this review could not find any direct measurement data of the metabolic heat generation rate of breast tissue, those of breast tumour tissue were studied by Gautherie [32]. This study clearly shows an inverse relation between the metabolic heat generation rate and the tumour volume doubling time. Ng et al. [39] also referred to this relation and used an equation relating the doubling time to the tumour diameter, while citing an earlier paper by Gautherie. Thus, most modelling studies used these equations to calculate the metabolic heat generation rate depending on the tumour size considered in their model.

The blood perfusion rate in the BHE is the only heat sink term. Heat is transferred by convection through the blood vessels in the tissue. Particularly in tumours, the chaotic and hypoxic environment makes the blood perfusion a complex system. Four studies in this review measure the blood perfusion through healthy and cancerous breast tissues using a thermal approach and PET imaging. In each study, the tumour blood perfusion was significantly higher than that of healthy tissue. While the study by Johnson [53] used a thermal approach to measure the blood perfusion rate, the measurement method proved unreliable. The remaining three studies conducted using PET imaging all have similar healthy to cancerous blood perfusion ratios. However, a large range of values for the measured tumour blood perfusions were observed in the three studies, where tumour blood perfusion measurements were not always higher than those of healthy breast tissue. Furthermore, Wilson et al. [56] noted that there could be no association made between blood perfusion and tumour size within their study.

Due to the lack of measurement data available, more recent thermal studies approximated the values for the thermal and physiological properties with those of other well-measured tissues. Glandular tissue properties were most often approximated from those of muscle tissue or thyroid gland, while fatty breast tissue properties were approximated from those of generic fat. In many cases, authors refer to a previous study in which an approximation has been made, who refer to another previous study, and so on. These trails lead to properties being approximated from values in eye muscle, such as in Zastrow et al. [47]. Another example is in Chanmugam et al. [41], where the tumour properties can be traced back to those of a skin lesion in [80]. Studies such as Converse et al. [36] provided too many references for all tissues used in the thermal model, without detailing how each individual property was obtained. This can be either difficult or impossible to trace back to the primary source of information. In such a case, the most likely scenario is that an average of the cited values was considered.

Certain studies also approximate some of the glandular and tumour properties to be the same. This can be seen in studies [33,39,41,43,46,47]. This may prove to be useful in thermal studies where the aim is to investigate the focusing ability of a hyperthermia system without relying on a thermal contrast already present. However, such approximations might provide unreliable results when considering preclinical scenarios and hyperthermia planning, especially considering the contrasting values presented in this review.

To summarise the data from this review, Table 7 presents the minimum and maximum values for each property of the fibroglandular breast, fatty breast, and breast tumour tissues. A general pattern emerges where the thermal and physiological properties of fat tissue are the lowest, tumour tissue properties are the highest, and fibroglandular properties lie in between for each of the five properties. However, there is an overlap of data, especially when comparing the thermal properties of fibroglandular and fatty breast tissue. This overlap arises from the heterogeneity of the breast tissue, where some studies such as González [33], Converse et al. [36], and Bakker et al. [46] do not consider the adipose content within the breast, but only consider breast tissue as a whole.

A notable difference can be seen in the ranges of the metabolic heat generation rate and blood perfusion rate of tumour tissue when compared to healthy breast tissue. The metabolic heat generation rate of tumour tissue was most often calculated from Equations (14) and (15). The lowest value of 690 W/$m^{3}$ is derived from Zastrow et al. [47], where the authors considered the properties of tumour tissue to be the same as those of glandular breast tissue. The range of the tumour blood perfusion rate is also much larger than those of fibroglandular and fat breast tissue. This is possibly due to the increased vascularisation via angiogenesis typical of tumours, which presents a chaotic system of new blood vessels that supply the tumour with oxygen [82].

While this review found tumour blood perfusion to be significantly higher than that of healthy tissue, this might not portray the reality. The vascularisation within a tumour changes as the tumour grows. The initial vascular system of the tumour can become compressed when the tumour starts to grow. This causes a reduction in the supply of oxygen and nutrients to the tumour, leading to a hypoxic environment, which is common in larger tumours that present a necrotic centre. Neovascularisation, the formation of new blood vessels, occurs within the tumour from pre-existing vessels or from the surrounding vessels within the healthy tissue. The efficiency of these new microvessels depends on how quickly they are formed, with rapidly growing tumours forming less structurally sound systems. As a result, the tumour blood perfusion may be more complex than the appearance of the tumour vascular system [83].

The blood perfusion rates of human tumours do not necessarily decrease with increasing tumour size [83]. Furthermore, a study by Shibata [84] found that areas surrounding the tumours have higher perfusion rates than the central tumour. The same study reported that the ratio of blood perfusion within healthy tissue to tumour tissue (not breast tumours) ranged from 3:1 to 30:1, implying that tumour blood perfusion may actually be lower than normal tissue blood perfusion. An overlap within healthy and tumour tissue blood perfusion was also seen in the data from this review, as shown in the data ranges presented in Table 7. The tumour blood perfusion also varies depending on the functional state of the breast tissue. The tumour within post-menopausal breast is substantially lower than the tumour within a lactating breast [83].

## 4. Reliable Data

In light of the various data sources and variability of the reported data presented in this review, both for measured data and estimated properties, it is clear that a consensus on the properties is required. We therefore present a set of values for the thermal and physiological properties of healthy and cancerous breast tissues in Table 8. The *Measured* data were analysed and studies with reliable measurement procedures were used to obtain these reference values. Where more than one study provided reliable data, the mean was considered with the minimum and maximum values noted in the table. These values obtained from measurement data are distinguished in boldface type within the table. For fibroglandular breast tissue, the review found reliable *Measured* data for the density ρ, effective thermal conductivity $k_{\mathrm{eff}}$, and intrinsic thermal conductivity $k_{\mathrm{int}}$. Reliable *measured* data were available for all the thermophysical properties of fatty breast tissue except for the metabolic heat generation rate, whilst reliable *measured* data for breast tumours were available for the specific heat capacity *c*, effective thermal conductivity $k_{\mathrm{eff}}$, intrinsic thermal conductivity $k_{\mathrm{int}}$, and blood perfusion rate $\omega_{t}$.

For density measurements, the studies by Woodard and White [31] and Johns and Yaffe [37] were excluded from this table. The former study did not directly measure the density of fibroglandular breast tissue, but calculations were made from the densities of the components in the tissue and the corresponding mass percentages. Although the latter study of Johns and Yaffe [37] did involve direct measurements of the density, these were only carried out after the tissue samples were frozen and thawed twice. Such a process is bound to cause a change in the water content of the tissue, and hence, these results were discarded in the calculation for Table 8.

The study by Robinson et al. [57] is the only study to present measured data on the specific heat capacity of breast tumours, which is a measurement conducted on breast carcinoma of the male breast.

The in vivo thermal conductivity measurements are affected by the blood perfusion, and as such, care should be taken when considering these values for modelling scenarios. Hence, measurements carried out in vivo and in vitro are separated into two categories: effective thermal conductivity ($k_{\mathrm{eff}}$) and intrinsic thermal conductivity ($k_{\mathrm{int}}$), respectively. For thermal conductivity calculations, that of mucinous carcinoma (0.350 W/m/K) presented by Gautherie [32] is also not included. This value was only quoted in the study within a discussion, and no indication was given on whether the value was obtained from conducting measurements. The study by Bowman [35] is considered with the data for the thermal conductivity of fibroglandular and tumour tissue. While the measurement method for human tissue measurements was not specifically stated, the same study describes a measurement method used on canine and rat tissue. As for Valvano et al. [62], the thermal conductivity of breast tumour at 37 ${}^{\circ }C$ was considered for the calculation of ($k_{\mathrm{int}}$).

No measurement data for the metabolic heat generation were found in this review. As for the blood perfusion rates, the early study by Johnson [53] was not used in the mean $\omega_{t}$ presented in Table 8. The measurement method consisted of measuring the heat flux across the patient’s skin in the presence of a heat sink. This heat sink was kept at different temperatures when measurements were conducted, which the author noted influenced the blood perfusion rates. The data from the study were difficult to extract, since the author gave conflicting units of measurement for the measured heat flux. While data from one patient were obtained, this was only possible since the original measurement data had been converted by a data analysis program that is not available digitally. As a result, the study was not considered reliable to be included in our references. However, the three measurement campaigns by Beaney [55], Wilson et al. [56] and Mankoff et al. [59] conducted through PET scans were used for the compiled data. Table 8 reports the mean value from these measurements together with the minimum and maximum values out of the three studies.

Where reliable *Measured* data were not available, approximations were required and are distinguishable in the table by the italicised font. A weighted average of the *Non-Measured* data was obtained, where the weight given to each estimated value was equal to the number of times the value was used within all literature found in this review. This accounts for the papers discussed in Section 3.6 and also the studies listed in the *Cited by* column in Table 3, Table 4 and Table 5. For instance, Table 3 lists four different values for the metabolic heat generation rate $q_{m}$ of fibroglandular breast tissue. The value 700 W/$m^{3}$ was used in a total of four studies [39,41,42,44], 450 W/$m^{3}$ used in three studies [33,40,45], 690 W/$m^{3}$ in another three studies [47,48,49], and 2092 W/$m^{3}$ in the remaining study [34]. Therefore, the approximated $q_{m}$ for fibroglandular breast tissue was calculated as follows:

$$q_{m,\mathrm{fib}}=\frac{\left(700\times 4)+(450\times 3)+(690\times 3)+(2092\times 1\right)}{11}=\;622\;W/m^{3}$$

This approach was also used for the specific heat capacity of fibroglandular tissue and the metabolic heat generation rate of fatty breast tissue. The metabolic heat generation rate of tumour tissue varies greatly and was most often approximated by Equations (14) and (15). Therefore, we recommend the use of these two equations based on measurement data for numerical studies involving a breast tumour of known diameter.

The only measurement data for the density of breast tumours found in this review were those by Johns and Yaffe [37], which describes a dubious measurement procedure. Therefore, the tumour density was attributed the same value as that of fibroglandular tissue. This assumption was made in light of the fact that most breast tumours form within the fibroglandular tissue [17] and the fact that the ESHO benchmarks assume a generic tumour density equal to that of muscle tissue (1090 kg/$m^{3}$) [70].

Similarly, no measurement data for the blood perfusion rate of fibroglandular breast tissue were found throughout the review. While approximations and estimations were made throughout literature, the value listed in Table 8 is the same as that of fatty breast tissue. We recommend the same blood perfusion rate within fatty and fibroglandular breast tissue since the measurement studies do not distinguish between the fat content, but rather, show the variation of blood perfusion between healthy and cancerous breast tissues.

## 5. Conclusions

This review presents the available data on the thermal and physiological properties of human breast tissue and breast tumours. These properties are the density ρ, specific heat capacity *c*, thermal conductivity *k*, metabolic heat generation rate $q_{m}$, and blood perfusion rate $\omega_{t}$. Such data are vital when modelling the temperature in breast tissue in thermal therapies such as microwave hyperthermia, for both computational studies and phantom experiments that should use tissue-mimicking properties. These properties are frequently used when considering thermal models such as the bioheat equation. This review considers data obtained from direct measurements of the properties and also approximations taken throughout the literature.

The available measured data on all the properties are lacking and are not always utilised in thermal models since they are not well known. The various methods of measuring the properties and unreliable experimental procedures suggest that further investigation into these properties is necessary. Such research ensures accurate and reliable modelling for thermal therapies, which rely on computer simulations to test the thermal therapy devices and treatment-planning platforms. The lack of accessible measurement data for the properties of healthy and cancerous breast tissue lead to authors relying on assumptions and approximations, which are necessary, but might not always be well sustained. In addition, each reference or calculated tissue property should be thoroughly documented to guarantee traceability.

In this review, we propose a reliable set of values for the thermophysical properties of healthy and cancerous breast tissues. These were obtained following the extensive review and a thorough scientific evaluation of the different measurement, estimation, or approximation methods. Thus, the proposed data set is a reliable and realistic one, which can be used for thermal simulations of the breast. Going forward, models and directives from the hyperthermia community (e.g., [70]) should be implemented to promote standardisation in modelling of breast cancer thermal therapies. Furthermore, this allows the alignment of the diagnostic criteria already adopted in order to give a more informative support to the performance of breast hyperthermia treatments.

## Figures and Tables

**Table 1:** Conversion table for units of blood perfusion rate.

Units of Blood Perfusion Rate	→	Units of Blood Perfusion Rate
mL/min/kg	$\times \frac{10^{-6}\rho_{b}\rho }{60}$	kg/s/$m^{3}$
mL/min/100 g	$\times \frac{10^{-6}\rho_{b}\rho }{6}$	
mL/min/g	$\times \frac{10^{-4}\rho_{b}\rho }{6}$	
mL/min/100 mL	$\times \frac{\rho_{b}}{6000}$	
mL/min/dl		
kg/min/kg	$\times \frac{\rho }{60}$	
mL/s/mL		
$m^{3}$/s/$m^{3}$	$\times \rho_{b}$	
/s		

**Table 2:** Quantities considered for converting blood perfusion rate units to the SI units. $\rho_{\mathrm{fib}}$, $\rho_{\mathrm{fat}}$, $\rho_{t}$, and $\rho_{b}$ are the density of fibroglandular breast, fatty breast, breast tumour, and blood, respectively. $c_{b}$ is the specific heat capacity of blood.

$\rho_{\mathrm{fib}}$	$\rho_{\mathrm{fat}}$	$\rho_{t}$	$\rho_{b}$	$c_{b}$
(kg/m${}^{3}$)	(kg/m${}^{3}$)	(kg/m${}^{3}$)	(kg/m${}^{3}$)	(J/kg/K)
1066.00	932.00	1066.00	1049.75	3622.50

**Table 7:** The minimum and maximum values of the thermal and physiological properties of healthy and tumour breast tissue.

Property	Fibroglandular Tissue		Fat Tissue		Tumour Tissue	
	Min.	Max.	Min.	Max.	Min.	Max.
$\rho \;(\mathrm{kg}/m{}^{3})$	920	1092	920	1080	920	1182
c(J/kg/K)	2493	3770	2220	3000	3000	3852
k(W/m/K)	0.255	0.500	0.120	0.385	0.280	0.594
$q_{m}\;(W/m{}^{3})$	450	2092	350	1180	690	65,400
$\omega_{t}$ (kg/s/m${}^{3}$)	0.189	0.754	0.014	8.798	0.530	22.260

**Table 8:** Proposed thermal and physiological properties of healthy and cancerous breast tissues. The minimum and maximum values of the mean values are also included.

Property	Fibroglandular Tissue			Fat Tissue			Tumour Tissue		
	Mean	Min.	Max.	Mean	Min.	Max.	Mean	Min.	Max.
ρ (kg/m${}^{3}$)	**1066**	**1040**	**1092**	**932**	**930**	**934**	*1066* ^a^	-	-
*c* (J/kg/K)	*3398*	*2493*	*3770*	**2220**	-	-	**3610** ^b^	-	-
$k_{\mathrm{eff}}$ (W/m/K)	**0.328**	**0.286**	**0.370**	**0.171**	-	-	**0.511**	-	-
$k_{\mathrm{int}}$ (W/m/K)	**0.359**	**0.255**	**0.499**	**0.165**	**0.120**	**0.209**	**0.442**	**0.280**	**0.564**
$q_{m}$ (W/m${}^{3}$)	*1180*	*450*	*2092*	*458*	*350*	*400*	*varies* ^c^	*690*	*65,400*
$\omega_{t}$kg/s/$m^{3}$)	*0.886* ^d^	-	-	**0.886**	**0.700**	**0.980**	**4.830**	**3.307**	**5.968**

N.B. **Boldface** values are the reliable values obtained from purely measurement data. *Italicised* values are those obtained from approximations and estimations. ^a^ Using the same value as the density of fibroglandular tissue. ^b^ Measured specific heat capacity of adenocarcinoma of the male breast. ^c^ Metabolic heat generation of breast tumours varies depending on the size of the tumour. This can be calculated using Equations (14) and (15). ^d^ Using the same value as the blood perfusion rate of fatty breast tissue.

## Data Availability

Not Applicable.

## Abbreviations

The following abbreviations are used in this manuscript:

BHE	Bioheat equation
HT	Hyperthermia therapy
IDC	Infiltrating ductal carcinoma
ILC	Infiltrating lobular carcinoma
MRI	Magnetic resonance imaging
MW	Microwaves
PET	Positron emission tomography
RF	Radiofrequency
SAR	Specific absorption rate
